# Impact of COVID-19 restrictions on incidence of gastrointestinal protozoal infections in Mexico and their association with environmental and socioeconomic risk factors

**DOI:** 10.1051/parasite/2025049

**Published:** 2025-08-19

**Authors:** Lissethe Palomo-Ligas, Filiberto Gutiérrez-Gutiérrez

**Affiliations:** 1 Facultad de Ciencias Químicas, Universidad Autonoma de Coahuila, Unidad Saltillo Saltillo Coahuila 25280 México; 2 Departamento de Farmacobiología, Centro Universitario de Ciencias Exactas e Ingenierías, Universidad de Guadalajara Guadalajara Jalisco 44430 México

**Keywords:** Epidemiology, Disease prevention, Public Health Policies, Protozoa, Pandemic effects, Parasite incidence

## Abstract

Gastrointestinal infections caused by protozoan parasites remain a significant public health concern worldwide, particularly during the health crisis caused by the COVID-19 pandemic, which led to severe economic and social crisis that highlighted the inadequacy of healthcare services in many countries. In this study, we analyzed changes in the incidence of cases of amebiasis, giardiasis, and other gastrointestinal protozoal infections before (2017–2019) and during (2020–2022) the pandemic. Our findings indicate a decrease in the incidence of these infections, with no significant variations in incidence by gender or age, and a higher incidence during months with elevated temperature and humidity. Sociodemographic factors, including residence in homes with earthen floors, poverty, limited access to healthcare services, inadequate nutrition, unemployment, and overcrowded living conditions, were associated with an increased risk of infection. Additionally, our results highlight the impact of public health policies on disease control, demonstrating that COVID-19 containment measures – such as international travel restrictions, workplace closures, event cancellations, stay-at-home mandates, and enhanced hand hygiene – also contributed to reducing parasitic infections. The persistent prevalence of protozoal infections in both periods underscores the urgent need to improve sanitation, personal hygiene, and public health education, particularly in developing countries, to mitigate their high burden.

## Abstract (fr)

Les infections intestinales causées par des protozoaires parasites demeurent un problème de santé publique majeur dans le monde entier, en particulier pendant la crise sanitaire provoquée par la pandémie de COVID-19, qui a entraîné une grave crise économique et sociale mettant en évidence l’insuffisance des services de santé dans de nombreux pays. Dans cette étude, nous avons analysé l’évolution de l’incidence des cas d’amibiase, de giardiase et d’autres infections gastro-intestinales à protozoaires avant (2017-2019) et pendant (2020-2022) la pandémie. Nos résultats indiquent une diminution de l’incidence de ces infections, sans variations significatives selon le sexe ou l’âge, et une incidence plus élevée pendant les mois où la température et l’humidité sont élevées. Des facteurs sociodémographiques, notamment le fait de vivre dans un logement au sol en terre battue, la pauvreté, un accès limité aux services de santé, une alimentation inadéquate, le chômage et le surpeuplement, étaient associés à un risque accru d’infection. De plus, nos résultats soulignent l’impact des politiques de santé publique sur la lutte contre la maladie, démontrant que les mesures de confinement liées à la COVID-19 – telles que les restrictions de voyages internationaux, la fermeture des lieux de travail, l’annulation d’événements, le confinement et le renforcement de l’hygiène des mains – ont également contribué à réduire les infections parasitaires. La prévalence persistante des infections à protozoaires au cours des deux périodes souligne l’urgence d’améliorer l’assainissement, l’hygiène personnelle et l’éducation en santé publique, en particulier dans les pays en développement, afin d’atténuer leur lourd fardeau.

## Introduction

Gastrointestinal (GI) parasitic infections caused by protozoa are among the most common infectious diseases worldwide [23]. According to the World Health Organization (WHO), GI parasites are responsible for approximately 67.2 million infections annually [19, 44]. Among the protozoa of greatest epidemiological significance, *Entamoeba histolytica* and *Giardia lamblia* [45] are responsible for amebiasis and giardiasis, respectively. These diseases are characterized by nausea, vomiting, diarrhea, fatigue, weight loss, anorexia, and abdominal pain, among other symptoms [16]. These infections are primarily transmitted through the ingestion of water and food contaminated with feces, with epidemiological studies reporting a higher prevalence in developing countries, particularly those with warm and humid climates [12]. Another intestinal protozoan of medical and zoonotic relevance is *Balantidium coli*, which can cause bloody diarrhea and, in some cases, lead to extraintestinal infection such as peritonitis, urogenital tract involvement, and pulmonary inflammation [15]. Additionally, *Cryptosporidium* spp. is a significant enteric parasite responsible for diarrhea and environmental enteric dysfunction, contributing to malnutrition and growth impairment [33]. Additionally, sociodemographic factors such as lack of access to clean water, inadequate sanitation and hygiene, extreme poverty, malnutrition, overcrowding, limited knowledge of transmission routes, and insufficient epidemiological surveillance contribute to a high incidence of these parasitic infections [26], mainly affecting children and immunocompromised individuals [5, 16].

On December 31, 2019, the WHO reported the emergence of a coronavirus strain (SARS-CoV-2) that causes a severe and potentially lethal disease known as COVID-19 [7]. This virus has a high infectivity rate and spread rapidly worldwide, generating a global health crisis [2], which led the WHO to declare it a pandemic on March 11, 2020 [8]. The rapid increase in COVID-19 cases worldwide compelled health authorities in countries to implement stringent containment measures, including border closures, restrictions on human mobility, and limitations on commercial activities [23]. Additionally, public health recommendations emphasized mask use, frequent handwashing, avoiding face-touching, disinfection practices, and social distancing [23]. These measures were crucial to prevent the spread of COVID-19. However, health systems shifted their focus toward COVID-19 care and prevention, which had direct consequences on the management of other diseases. This included a significant decrease in hospital visits and medical examinations, but also an increase in health control measures [8, 32].

Given the profound healthcare disruptions caused by the pandemic, understanding its impact on GI infections caused by parasitic protozoa is crucial. Mexico represents a particularly relevant case, as it was among the countries most affected by COVID-19, while also exhibiting a high rate of GI protozoal infections. Moreover, Mexico is a middle-income country with a consolidated healthcare system that mandates the notification of GI parasitic infections to regulatory entities [20]. In this study, we analyzed the national surveillance data of amebiasis, giardiasis, and other intestinal protozoal infections (balantidiasis, cryptosporidiosis, and unspecified protozoa), their changes during the COVID-19 pandemic, and their association with changes in public health policies, sanitation practices, and socioeconomic factors during the peak pandemic period.

## Materials and methods

### Period analyzed and case definition

Mexican health regulations, established by the Ministry of Health, through the Official Mexican Standards (NOM-032-SSA2-2010, NOM-017-SSA2-2012), mandate the nationwide weekly reporting of amebiasis, giardiasis, and other intestinal protozoal infections *via* the General Directorate of Epidemiology. Epidemiological morbidity data, categorized by federal entity, month, age, and sex for the period 2017–2022, were retrieved from the official database https://epidemiologia.salud.gob.mx/anuario/html/index.html.

Data on socioeconomic and environmental factors, including the relative change, with respect to pre-pandemic years (2017–2019), in the population without access to clean water, drainage, healthcare services, adequate nutrition, and quality food, as well as reductions in overcrowding, poverty, and households with earthen floors, alongside the percentage increase in unemployment from 2017 to 2022, were obtained from the National Council for the Evaluation of Social Development Policy (CONEVAL) at https://www.coneval.org.mx/Medicion/MP/Paginas/Pobreza_2022.aspx. Additionally, public health policies implemented for pandemic control in each federative entity were consulted from the study by Knaul *et al.* [23]. The Mexican territory is geographically divided into 32 states.

### Screening and data selection criteria

The data were categorized into two periods, pre-pandemic (2017–2019) and post-pandemic (2020–2022). Incidence rates (number of new cases of the disease per 100,000 inhabitants) of amebiasis, giardiasis, and other intestinal protozoal infections were analyzed by month, year, federal entity, and demographic variables (age group and sex) to assess changes between these periods.

National distribution maps were generated to illustrate the percentage reduction in average incidence of amebiasis, giardiasis, and other intestinal protozoal infections during the analyzed periods. Additionally, a choropleth map was constructed to represent the relative change, with respect to pre-pandemic years (2017–2019), in socioeconomic factors associated with the risk of amebiasis, giardiasis, and other intestinal protozoal infections, such as the reduction in the population without access to clean water, drainage, and health care services; reduction in overcrowding, poverty, and households with earthen floors; reduction in the population without access to adequate nutrition and quality food; and increase in the unemployed population. The values correspond to the percentage variation observed between to pre-pandemic years (2017–2019), and post-pandemic years (2020). This approach allowed for the identification of states where the socioeconomic factors deteriorated or improved in relative terms during the early stages of the COVID-19 pandemic, highlighting regional disparities that may have influenced vulnerability to intestinal infections caused by parasites.

To assess the influence of the different public policies implemented in each state of the Mexican Republic during the COVID-19 pandemic, data from Knaul *et al.* 2021 [23] were used. In this study, we evaluated specific indicators related to mobility restrictions and virus containment, such as school closures, closure of non-essential jobs, cancellation of public events, internal mobility restrictions, international travel restrictions, and stay-at-home measures.

### Statistical analysis

To analyze annual trends of intestinal protozoal infections in Mexico and assess the impact of COVID-19 containment measures, an autoregressive integrated moving average (ARIMA) model was applied. First, the time series was stabilized using a logarithmic transformation. Then, the autocorrelation function (ACF) and the partial autocorrelation function (PACF) were then computed to detect seasonal patterns. Subsequently, an ARIMA model was used to analyze autocorrelations and forecast future trends, as shown in Equation (1):

(1) $$\mathrm{ARIMA}\;=\;(p,\;d,\;q){\left(P,\;D,\;Q\right)}_{m}$$

where:

*p* = number of lagged values in the non-seasonal component.
*d* = order of non-seasonal differences.
*q* = order of lagged errors in the non-seasonal component.
*P* = order of seasonal lags.
*D* = seasonal differentiation.
*Q* = order of lagged errors in the seasonal component.
*m* = number of time series observations in a seasonal cycle.

To compare the average incidence of amebiasis, giardiasis, and other intestinal protozoal infections between the pre-COVID-19 and post-COVID-19 periods, a T-test for variance analysis was conducted using Real Statistics software in Excel. Additionally, an analysis of variance (ANOVA) was performed to determine statistically significant differences (*p* < 0.05) across time, considering year, month, sex, and season.

To explore associations between environmental and socioeconomic variables in each state with the incidence of intestinal protozoal infections, data on the average percentage variation observed in Mexico between the pre-pandemic years (2017–2019) and the post-pandemic year (2020) were used. An additional T-test for variance analysis was conducted using Real Statistics software in Excel, with statistically significant differences at *p* < 0.05. All statistical analyses were performed using GraphPad Prism version 6.01 for Windows.

## Results

### Annual comparison of protozoal disease cases: pre-pandemic (2017–2019) *vs.* post-pandemic (2020–2022)

**Figure 1:**
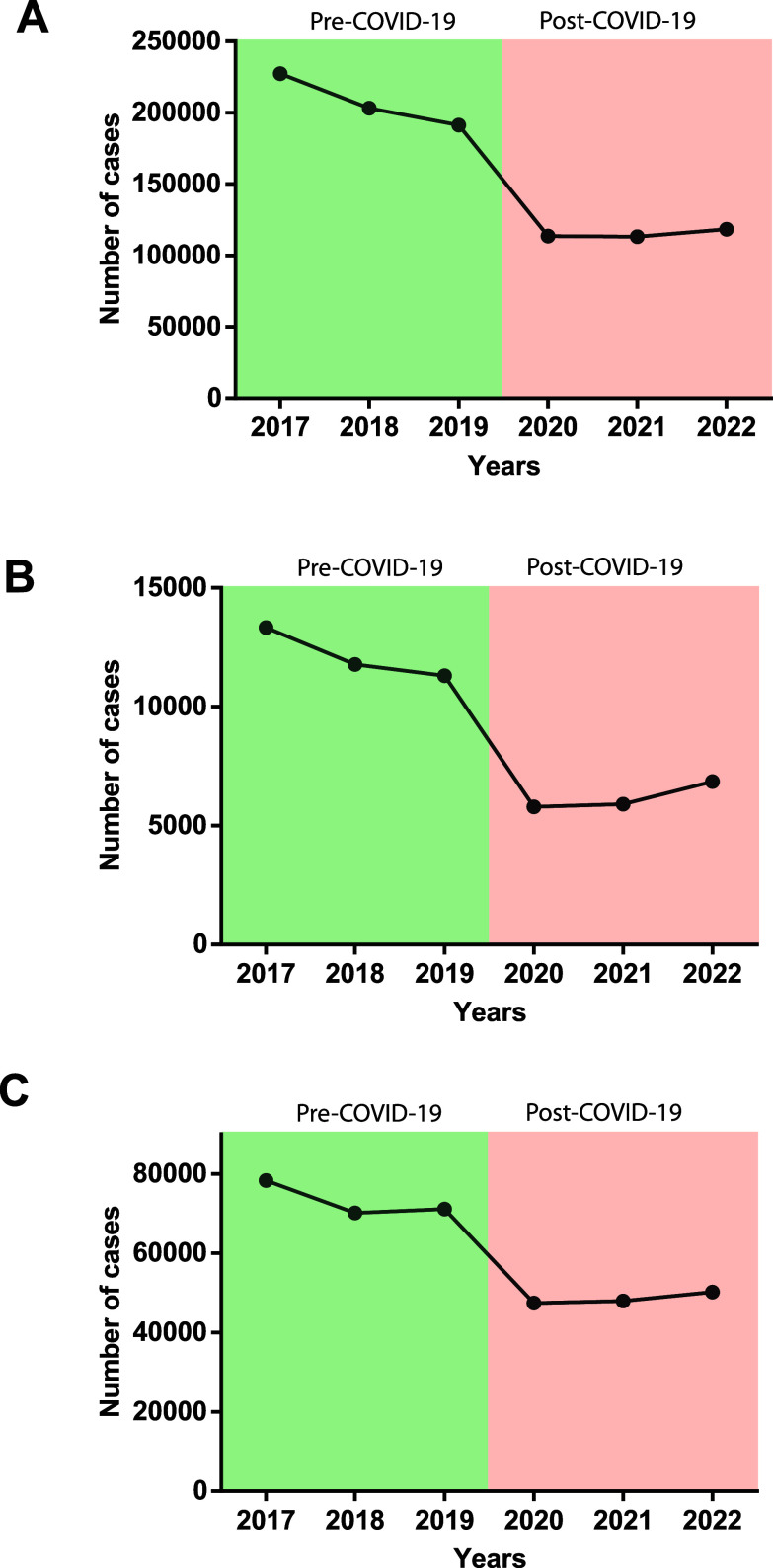
Number of cases of (A) amebiasis, (B) giardiasis, and (C) other intestinal protozoal infections in Mexico. The pre-pandemic period (2017–2019) is in green, while the post-pandemic period (2020–2022) is in red. Data analysis was performed using GraphPad Prism version 6.01 for Windows, and differences were considered statistically significant at *p* ≤ 0.001.

The data analysis revealed a clear downward trend in the number of cases of intestinal protozoal infections in the post-pandemic period compared to pre-pandemic levels. During 2017–2019, the average annual cases of amebiasis, giardiasis, and other intestinal protozoal infections were 20,730, 12,140, and 73,214, respectively. In contrast, during 2020–2022, the cases dropped significantly to 11,075, 6,184, and 48,559, respectively reflecting reductions of 44.5% (amebiasis), 49.06% (giardiasis), and 33.78% (other protozoal infections) (Fig. 1).

Statistical analysis confirmed a significant difference between pre-COVID-19 and post-COVID-19 case numbers. The most pronounced decline occurred in 2020, coinciding with the strictest COVID-19 preventive measures. However, as restrictions eased in 2021 and 2022, a slight rebound was observed, with case increases of 4.12% (amebiasis), 18.26% (giardiasis), and 5.82% (other intestinal protozoal infections) between 2020 and 2022 (Fig. 1). A statistically significant decrease in incidence was observed for all protozoal infections when comparing the pre-pandemic and post-pandemic periods (Table 1).

**Table 1:** Changes in the incidence of amebiasis, giardiasis, and other GI protozoal infections in Mexico during the pre-pandemic period (2017–2019) and post-pandemic period (2020–2022). The data were analyzed using GraphPad Prism version 6.01 for Windows, and differences were considered statistically significant at *p* ≤ 0.001.

	Mean number of cases		Percent reduction in cases	*p*-value
	Pre-COVID-19	Post-COVID-19		
Amebiasis	198,88	100,34	49.55	<0.0001
Giardiasis	11,56	5,49	52.51	0.0009
Other protozoa	66,04	42,09	36.27	0.0002

### Pattern of target GI infections during the COVID-19 pandemic (2020–2021)

**Figure 2:**
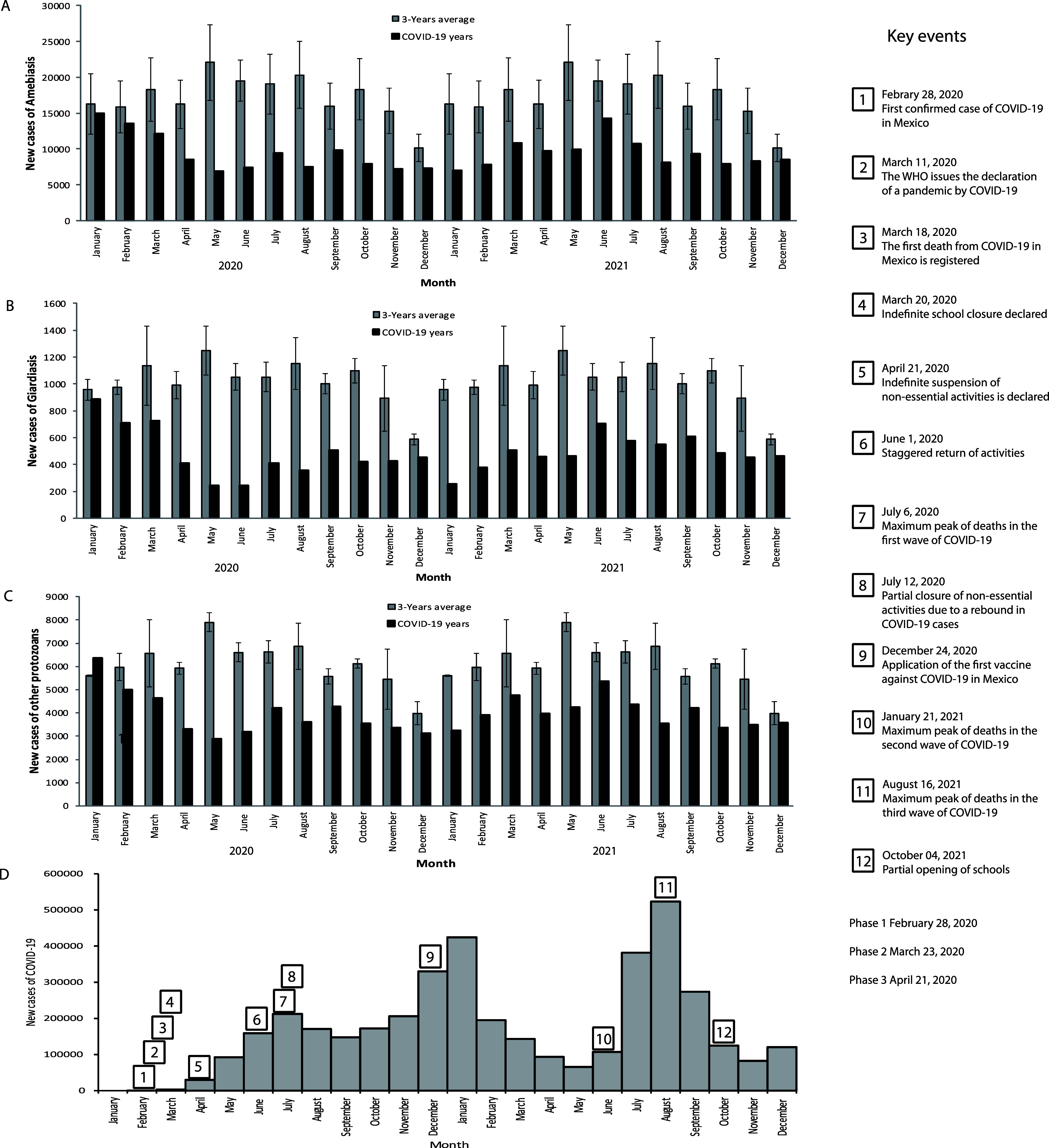
Changes in the pattern of GI infections caused by protozoan parasites during different phases of the COVID-19 pandemic (2020–2021). There are the new cases of A) amebiasis; B) giardiasis; and C) other intestinal protozoal infections. The grey bars represent the average monthly number of cases in the three years preceding the pandemic (2017–2019), while the black bars indicate the number of new cases reported during the pandemic years.

The analysis of GI infections caused by protozoan parasites during the different phases of the COVID-19 pandemic from 2020 to 2021 revealed significant changes in incidence, closely linked to public health measures and shifts in social behaviors (Fig. 2). At the beginning of 2020, a decrease in the number of reported cases of amebiasis and giardiasis was observed in January, although still with relatively high numbers, while in February an even greater decrease was observed in the number of reported cases of amebiasis, giardiasis, and other protozoal infections relative to the average monthly cases from the preceding three years. This downward trend became more pronounced in March and April 2020, coinciding with the World Health Organization’s official declaration of the COVID-19 pandemic and the implementation of Phase 1 measures in Mexico, including the indefinite closure of schools. During these months, the number of cases dropped considerably, with reductions of 33.06% and 46.87% for amebiasis, 36.2% and 58.63% for giardiasis, and 29.62% and 44.16% for other intestinal infections caused by protozoa.

The most significant decline occurred in May 2020, when stricter restrictions were introduced as Mexico entered Phases 2 and 3 of the pandemic. This period saw the closure of all non-essential businesses, a measure that coincided with the largest reductions in the number of reported infections, reaching 68.38% for amebiasis, 80.29% for giardiasis, and 63.51% for other protozoal infections. However, with the gradual reopening of commercial activities on June 1, 2020, there was an evident resurgence of COVID-19 cases, peaking in June and July. Relaxation of social isolation measures led to a rise in the number of GI infections caused by protozoan parasites, suggesting a possible relationship between increased mobility and exposure risks.

By mid-July 2020, the authorities had reinstated partial restrictions, leading to a slight decline in cases of COVID-19 in August. This period saw reductions of 62.47% for amebiasis, 68.97% for giardiasis, and 47.7% for other protozoal infections relative to the average monthly cases from the preceding three years. However, by September, cases of GI infections caused by protozoa began to rise again, followed by a phase of relative stability from October 2020 to January 2021.

In early 2021, the rollout of the first COVID-19 vaccines in Mexico led to the reopening of commercial activities and a relaxation of preventive measures. This coincided with an increase in GI infections caused by protozoa during February and March. As vaccination efforts progressed and by June 2021, when the most vulnerable populations had been immunized, the impact of the pandemic on protozoal infection rates appeared to lessen. However, the reductions observed were the smallest post-pandemic declines recorded, with amebiasis decreasing by 26.91%, giardiasis by 33.06%, and other protozoal infections by 18.93%.

**Figure 3:**
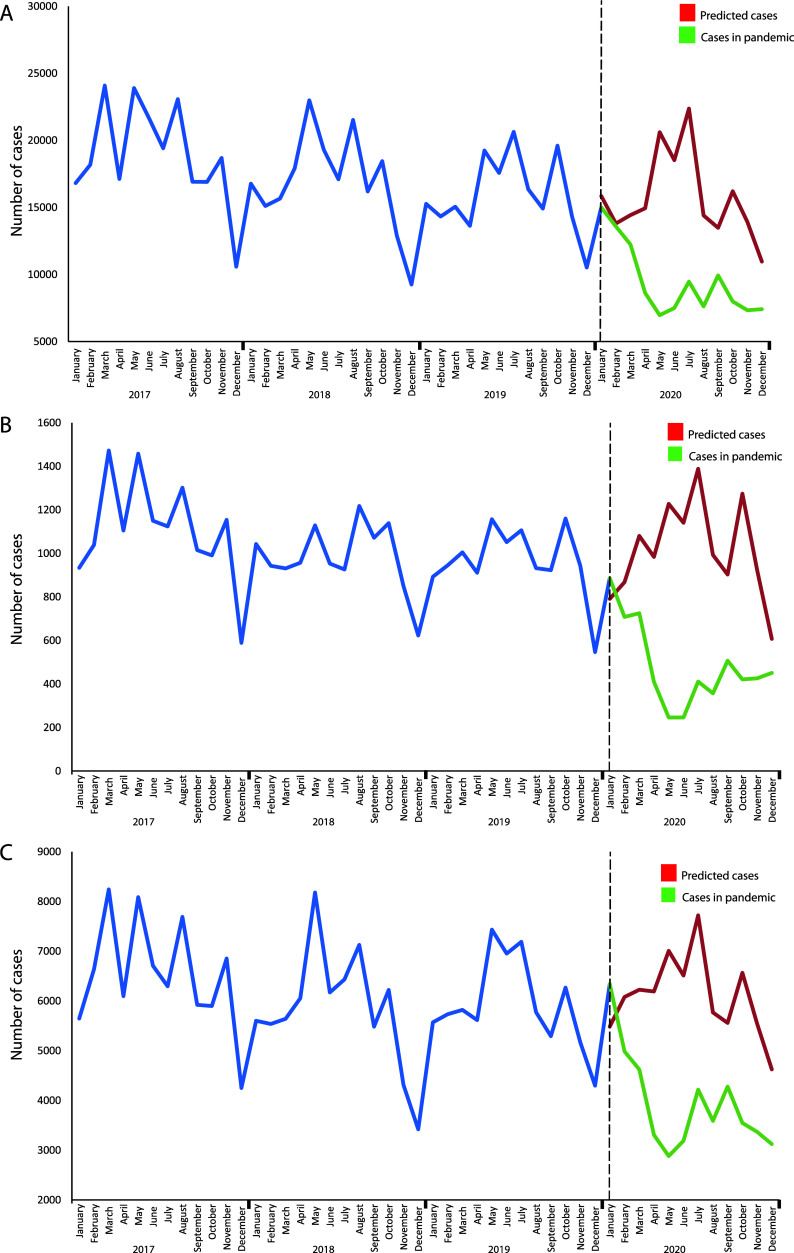
Autoregressive integrated moving average (ARIMA) model for GI protozoal infections at the onset of the COVID-19 pandemic in 2020. The predicted cases of the model (red) versus the actual reported (green). (A) amebiasis; (B) giardiasis; and (C) other intestinal protozoal infections. Statistical analyses were performed using Excel for Windows.

During the months of July and August 2021, a gradual decrease in cases of GI infections caused by protozoa was observed, followed by a period of stability, even after the reopening of schools in October of that year (Fig. 2). These data suggest that several factors, such as improved hygiene awareness and ongoing health measures, may have contributed to controlling transmission. To further analyze these trends, an ARIMA model was applied, comparing observed and projected data. The statistical analysis confirmed a significant decrease in the number of cases in 2020 when compared to the expected values based on previous years (Fig. 3). The ARIMA models for amebiasis (0,1,1)(1,0,0)_12_, giardiasis (0,1,1)(1,0,0)_12_, and other intestinal protozoal infections (1,1,2)(0,1,1)_12_ provided robust predictions supporting these findings. These results highlight the complex interplay between public health policies, pandemic-related restrictions, and the transmission of GI infections caused by protozoan parasites.

### Pattern of distribution by age and sex of GI infections due to the COVID-19 pandemic

**Figure S1.**
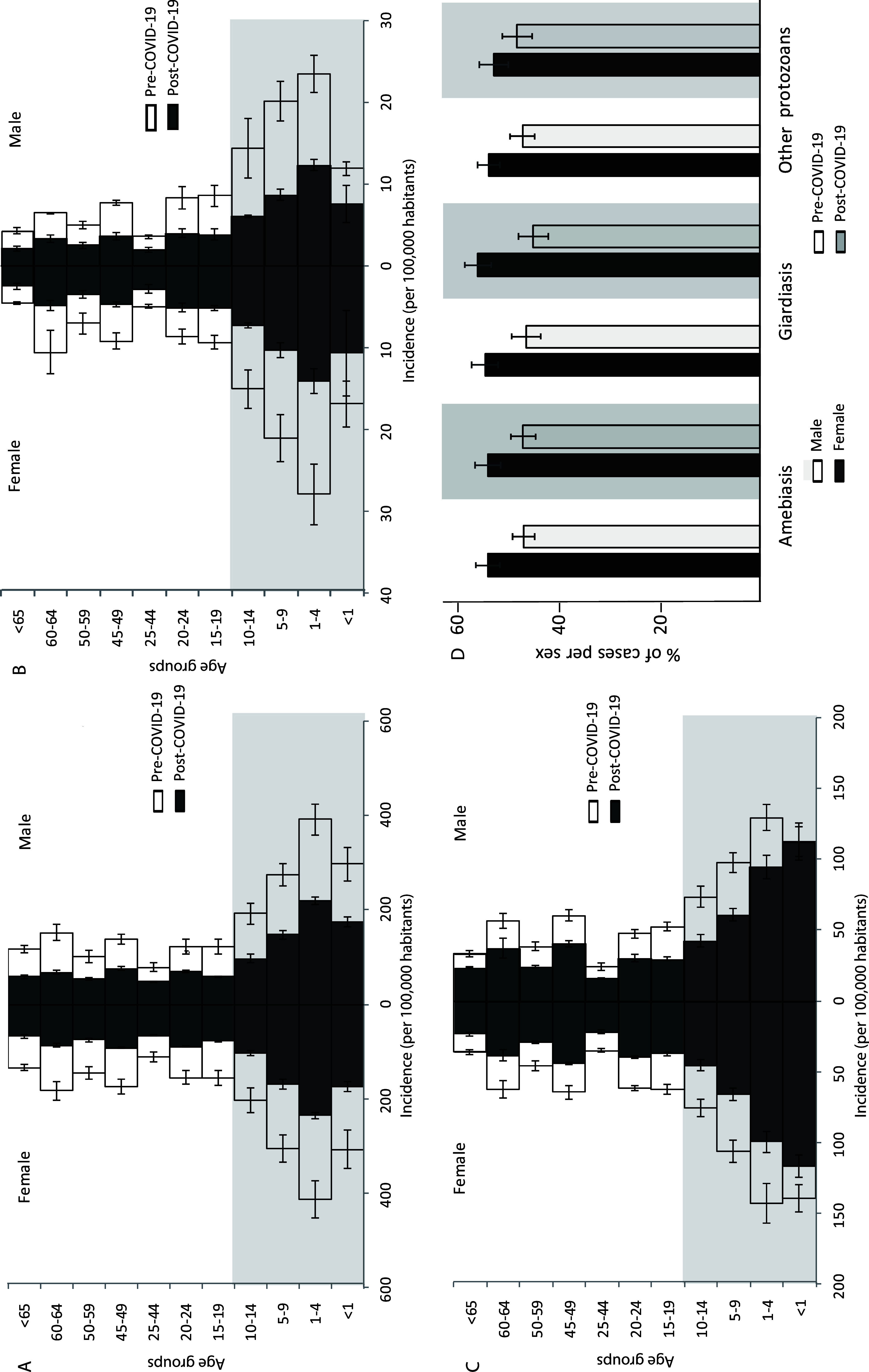
Analysis of changes in the age and sex distribution of GI infections caused by protozoan parasites in Mexico due to the COVID-19 pandemic. A) Incidence of amoebiasis; B) giardiasis; and C) other protozoal infections, with the most affected age group shaded in grey; D) distribution of cases by sex. In figure D, grey represents the average monthly cases of GI infections caused by protozoan parasites in the last three years pre-pandemic (2017–2019), while black represents the number of new cases of gastrointestinal infections caused by protozoan parasites during the COVID-19 pandemic (2020–2022). Statistical analyses were performed using GraphPad Prism version 6.01 for Windows, with significance set at *p* ≤ 0.05.

The analysis of age distribution patterns in GI infections caused by protozoan parasites during the COVID-19 pandemic showed no significant differences in the most affected age group between the pre-pandemic and post-pandemic periods. The highest incidence was consistently observed in children under 1 year of age and in the 1–4 and 5–9-year-old age groups. Among these, the 1–4-year-old population exhibited the highest number of cases throughout the analyzed period (Figs. S1A–S1C). Conversely, the lowest incidence of protozoal infections was recorded in individuals aged 25 to 44 years across all cases.

Similarly, the analysis of sex distribution patterns showed no significant changes in the proportion of infections between men and women before and after the pandemic. However, a higher incidence was consistently observed in female patients. The proportion of cases in women was as follows: giardiasis pre-pandemic (54.06 ± 2.62%) and post-pandemic (55.43 ± 2.55%), amebiasis pre-pandemic (53.48 ± 2.31%) and post-pandemic (53.45 ± 2.48%), and other protozoal infections pre-pandemic (53.34 ± 2.17%) and post-pandemic (52.29 ± 2.87%).

### Monthly distribution of amebiasis, giardiasis, and other intestinal protozoal infections due to the COVID-19 pandemic

**Figure 4:**
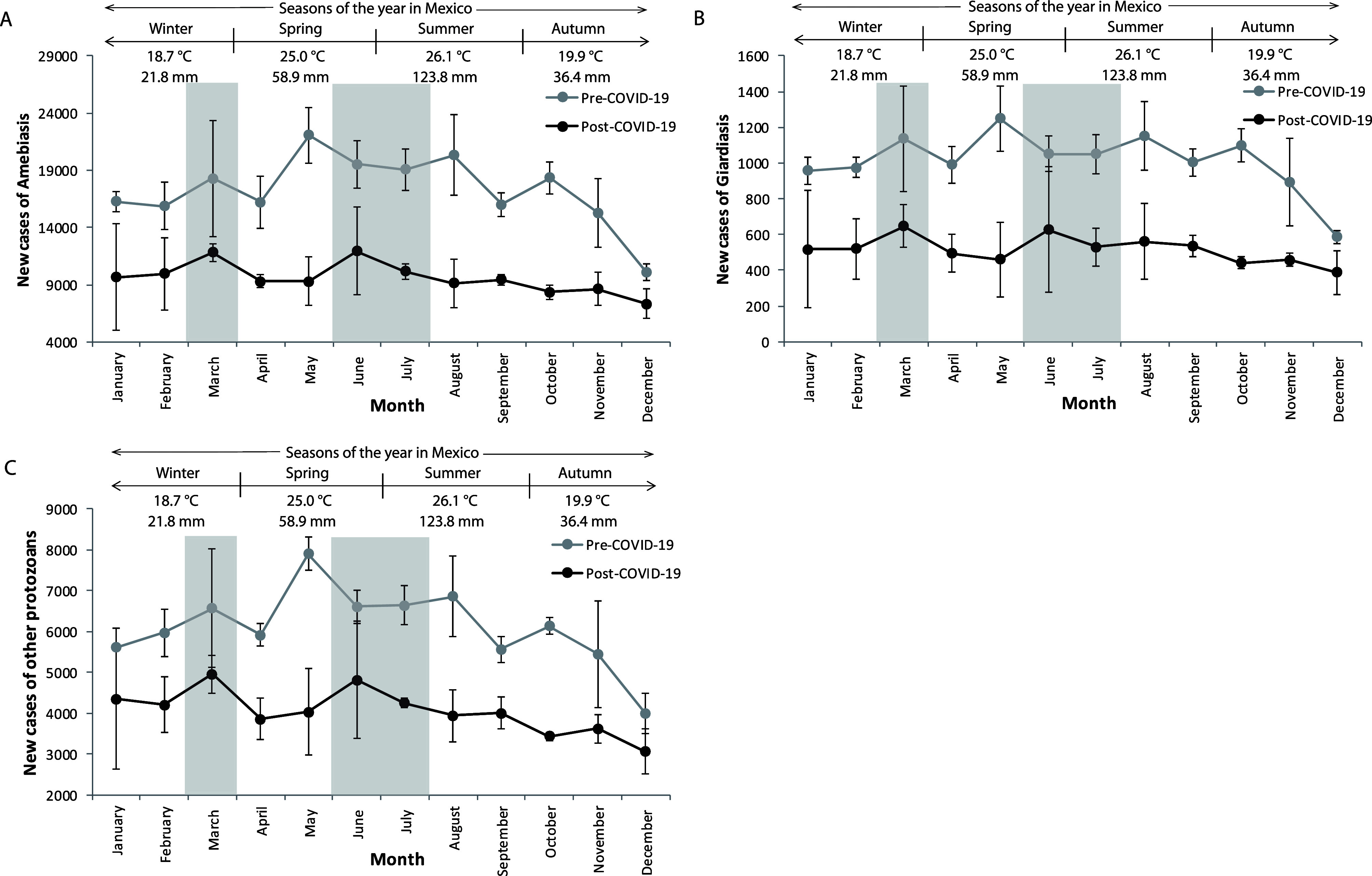
Seasonal variations in amoebiasis, giardiasis, and other intestinal protozoal infections in Mexico, in the pre-pandemic (2017–2019) and post-pandemic (2020–2022) periods. A) New cases of amebiasis; B) giardiasis; and C) other intestinal protozoal infections. The periods with the highest incidence correspond to the warm and humid months of spring and summer in Mexico (shaded in grey). Average temperature during the period 2019–2020 and precipitation during the year 2021. Data were analyzed using GraphPad Prism version 6.01 for Windows.

The monthly distribution of new cases was analyzed across the four annual seasons (winter, spring, summer, and autumn) for the pre-pandemic years (2017–2019) and compared with data from the pandemic years (2020–2022). In the pre-pandemic period, the highest incidence of amebiasis, giardiasis, and other intestinal protozoal infections occurred during the warm and humid months of May to August, aligning with spring and summer in Mexico (Fig. 4). Interestingly, the general seasonal patterns of infection continued in the post COVID era, only with overall reduced case numbers and a dampened peak during spring/summer.

Conversely, the lowest number of new cases in the pre-pandemic years was recorded during the colder, drier months of November to January (autumn and winter), a trend that persisted post-pandemic, with lower incidence observed in October, November, and December (Fig. 4). These findings highlight a direct relationship between increased environmental temperature and humidity and the seasonal distribution of amebiasis, giardiasis, and other protozoal infections.

### Incidence and geographic distribution of amebiasis, giardiasis, and other intestinal protozoal infections due to the COVID-19 pandemic

**Figure 5:**
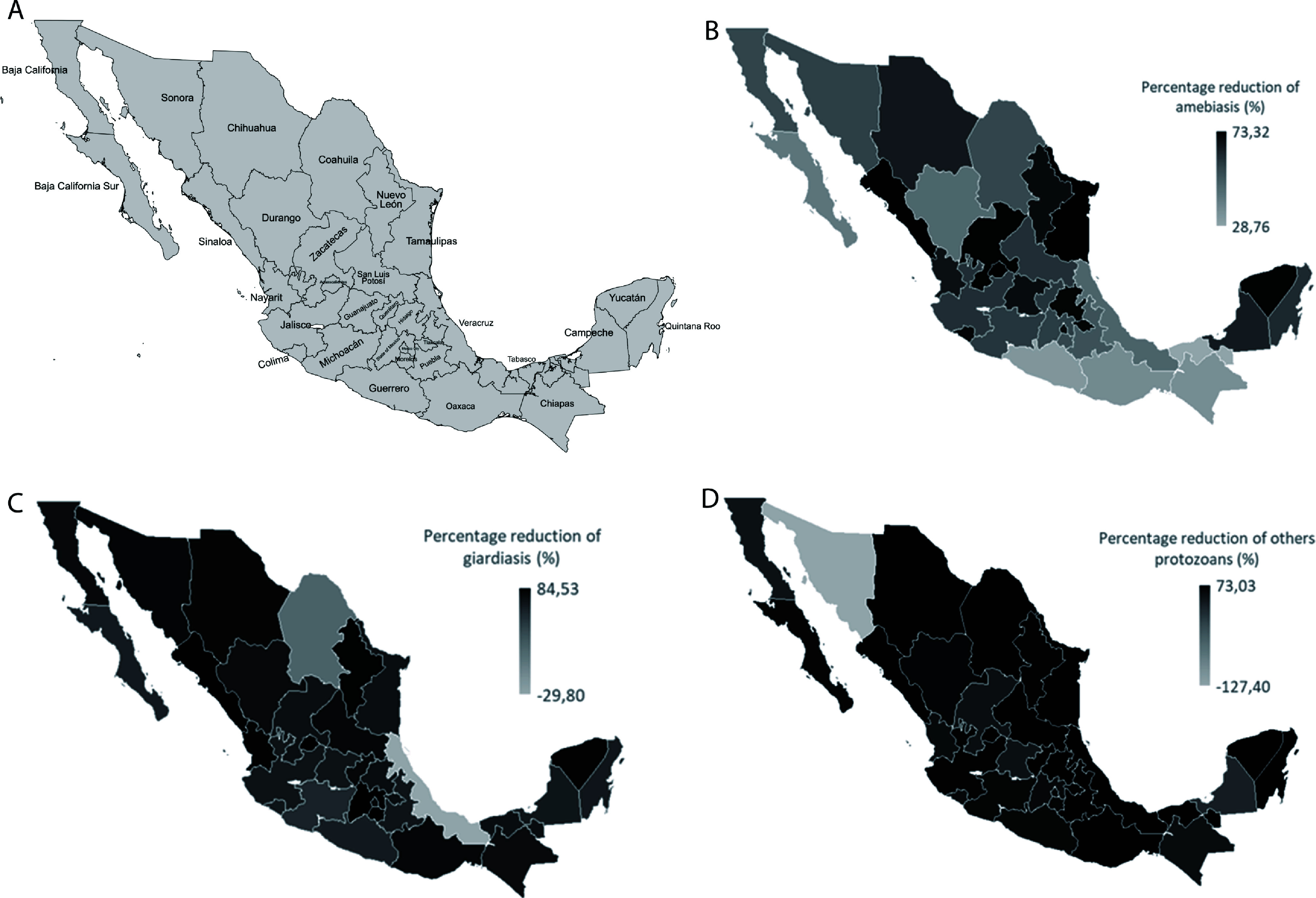
Changes in the incidence and geographic distribution of new cases of amebiasis, giardiasis, and other GI infections caused by protozoan parasites due to the COVID-19 pandemic. A) Distribution of coastal and non-coastal states; B) percent reduction in amebiasis cases by state; C) percent reduction in giardiasis cases by state; D) percent reduction in other parasitic protozoan cases by federal entity. In all maps, decreasing color intensity is used to represent the magnitude of reduction in infection incidence, with darker shades corresponding to more pronounced decreases.

To understand the changes in the incidence and distribution of new cases of amebiasis, giardiasis, and other intestinal infections caused by parasitic protozoa due to the COVID-19 pandemic, data on the average incidence by state in the pre-pandemic period (2017–2019) and the post-pandemic period (2020–2022) were analyzed. The relative change, with respect to the pre-pandemic period, as a percentage reduction for each parasitic infection was estimated. States with the greatest reductions in protozoal infection incidence varied by disease. For amebiasis, the most notable decreases were observed in Tamaulipas, Aguascalientes, Sinaloa, and Yucatán (Fig. 5B). Giardiasis incidence declined most significantly in Sinaloa, Mexico City, Morelos, and Aguascalientes (Fig. 5C), while other protozoal infections showed the largest reductions in San Luis Potosí, Yucatán, Sinaloa, and Coahuila (Fig. 5D).

**Figure 6:**
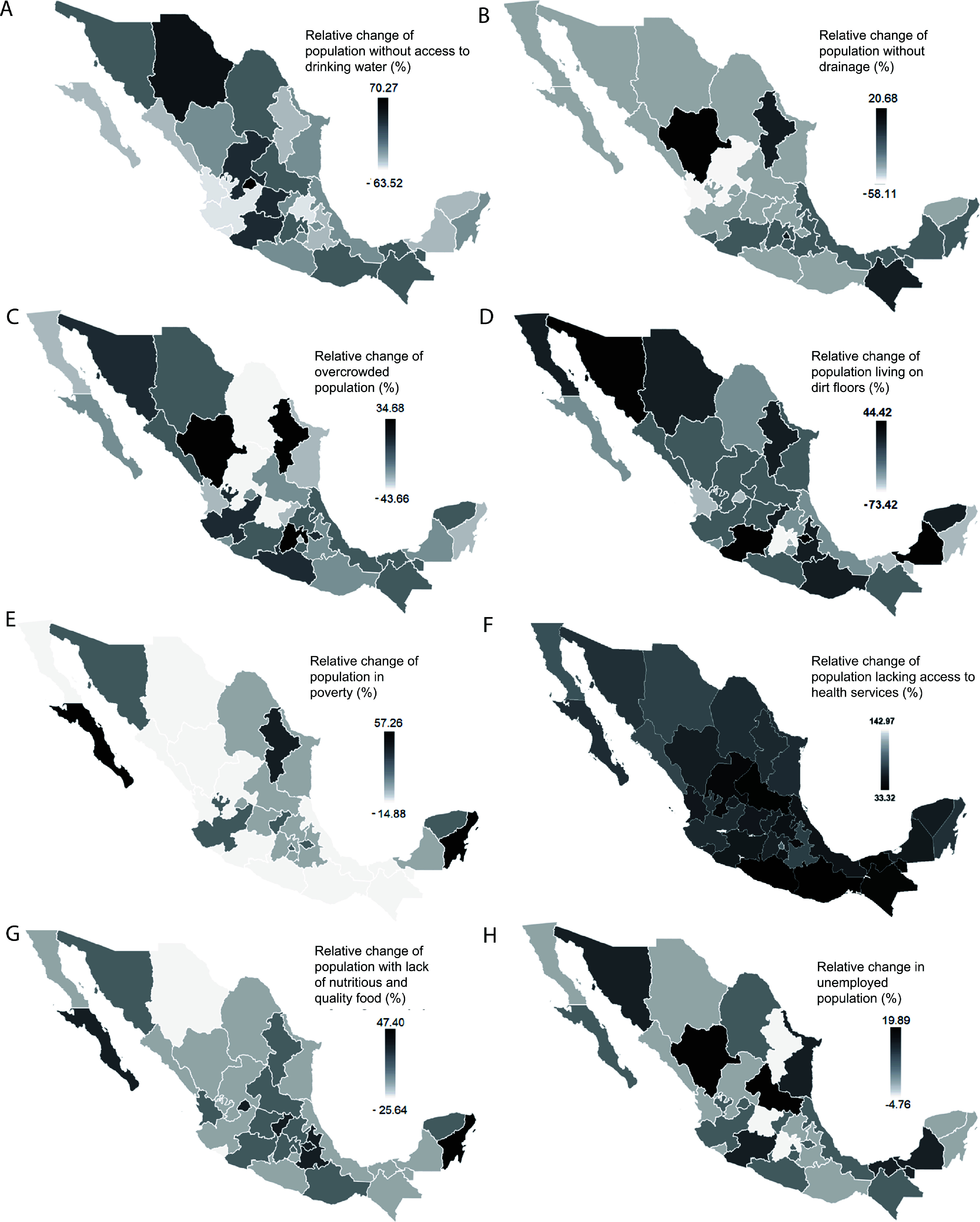
Maps illustrating changes in social development incidence associated with changes in the distribution of new cases of amebiasis, giardiasis, and other intestinal protozoal infections. A) Percent reduction in population without access to clean water; B) percent reduction in population without access to drainage; C) percent reduction in overcrowded population; D) percent reduction in population living with earthen floors; E) percent reduction in population living in poverty; F) percent reduction in population without access to health services; G) percent reduction in population without access to good nutrition and quality food; and H) percent increase in unemployed population. The color intensity gradient represents the magnitude of the analyzed factor.

A comparison of changes in social development indices in each state, in relation to distribution of new cases, reveals significant differences between the pre-pandemic period (2017–2019) and the post-pandemic period (2020–2022) (Table 2). According to Table 2, all analyzed variables – except two – significantly influenced changes in protozoan parasite incidence following the COVID-19 pandemic. However, further analysis revealed that Sinaloa, a state with relatively low poverty levels, exhibited one of the greatest reductions in protozoal infections (Fig. 6E), suggesting a possible indirect association. Similarly, although the overall impact of nutrition and food quality was not statistically significant, states with deficiencies in these areas tended to have higher protozoal infection rates (Fig. 6G), indicating that these factors may still contribute to parasite burden under certain conditions.

**Table 2:** Changes in social development associated with the pattern of distribution of new cases of amebiasis, giardiasis, and other intestinal infections caused by parasitic protozoa in the pre-pandemic period (2017–2019) and post-pandemic period (2020–2022). The data were analyzed using GraphPad Prism version 6.01 for Windows, and differences were considered statistically significant at *p* ≤ 0.001.

	Mean change in social and infrastructure deficits		*p*-value
	Pre-COVID-19	Post-COVID-19	
Percent reduction in population without access to clean water	7.04	6.30	0.,046
Percent reduction in population without access to drainage	6.25	4.86	0.0008
Percent reduction in overcrowded population	7.88	6.30	<0.0001
Percent reduction in population living with earthen floors	3.02	2.63	0.0144
Percent reduction in population living in poverty	40.60	42.17	0.0631
Percent reduction in population without access to health services	14.45	25.72	<0.0001
Percent reduction in population without access to good nutrition and quality food	22.51	22.85	0.6167
Percent increase in unemployed population	43.67	40.87	<0.0001

**Figure 7:**
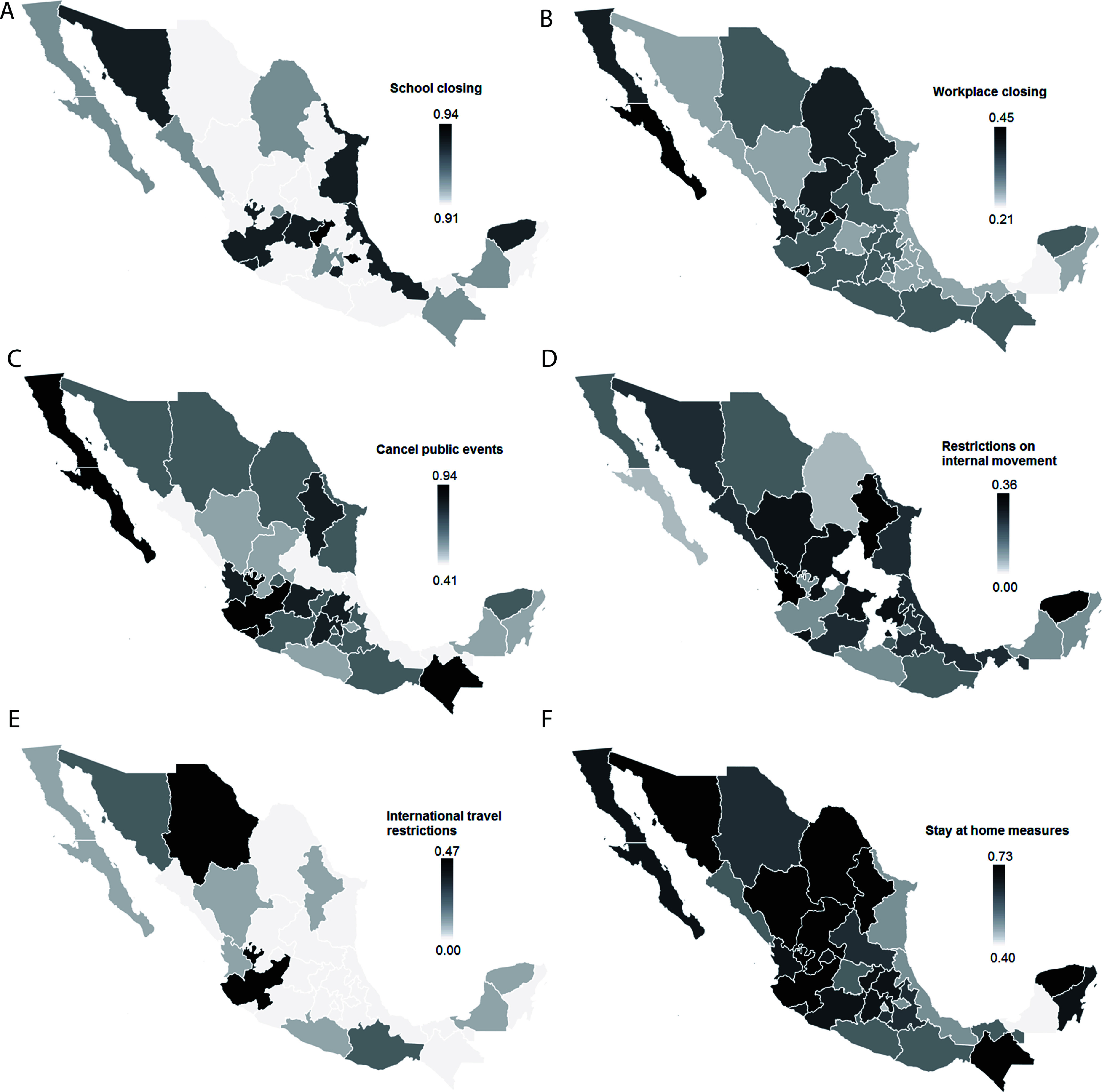
Maps depicting public health policies implemented to control the COVID-19 pandemic and their impact on the distribution of new cases of amebiasis, giardiasis, and other intestinal protozoal infections in Mexico. A) School closures; B) closure of non-essential jobs; C) cancellation of public events; D) internal mobility restrictions; E) international travel restrictions; and F) stay-at-home measures. The gradual increase in color intensity indicates the extent of the policy implemented.

Furthermore, states with greater reductions in parasitic infections, such as Yucatán, implemented more robust public health policies aimed at controlling the pandemic. Measures such as school closures (Fig. 7A), international travel restrictions (Fig. 7D), and stay-at-home orders (Fig. 7F) were notably more significant in these regions. Similar trends were observed in other states, including Coahuila (stay-at-home measures) (Fig. 7F), Morelos (school closures) (Fig. 7A), Mexico City (international travel restrictions) (Fig. 7D), and Aguascalientes (closure of non-essential businesses) (Fig. 7B).

The states with a lower reduction in new cases included Chiapas, Guerrero, Oaxaca, and Tabasco for amebiasis (Fig. 5B); Coahuila, Veracruz, Guerrero, and Michoacán for giardiasis (Fig. 5C); and Campeche, Baja California, Sonora, and Guanajuato for other intestinal infections caused by parasitic protozoa (Fig. 5D).

To contextualize these patterns, we examined changes in social development indicators and the intensity of public health interventions during the pandemic. In states with greater reductions, such as Yucatán, Mexico City, Morelos, and Aguascalientes, stricter containment measures were implemented. These included more frequent school closures (Fig. 7A), broader international travel restrictions (Fig. 7D), and more extensive business closures and stay-at-home orders (Figs. 7B, 7F).

In contrast, states with lower reductions in protozoal infections often exhibited socioeconomic vulnerabilities, including increases in the population living in overcrowded conditions (*e.g.*, Guerrero; Fig. 6C), reduced access to health services (*e.g.*, Tabasco, Oaxaca; Fig. 6F), and higher unemployment rates (*e.g.*, Campeche, Tabasco; Fig. 6H). Additionally, these states implemented fewer public health measures, such as school closures and event cancellations, and had lower enforcement of mobility restrictions (Figs. 7A–7F).

Together, these findings suggest that both structural inequalities and differences in pandemic response strategies contributed to the heterogeneous impact of COVID-19 on protozoal infection dynamics across states.

## Discussion

Considering the profound changes and challenges posed by the COVID-19 pandemic in healthcare, this retrospective study evaluated how health emergencies such as pandemics influence GI infections caused by parasitic protozoa, such as *E. histolytica* and *G. lamblia*, as well other protozoa-related infections (*Balantidium*, *Cryptosporidium* and unspecified intestinal protozoa) in Mexico. Additionally, the study examined the relationship between these infections and modifications in public health policies, sanitation measures, and socioeconomic conditions during the peak period of the COVID-19 pandemic.

Our analysis revealed a clear downward trend in the number of intestinal infections caused by parasitic protozoa in the post-pandemic stage compared to the pre-pandemic period with reductions of 33.78–49.06% in the analyzed parasites. The most significant reduction occurred in 2020, the first year of the pandemic, when COVID-19 preventive measures were at their strictest. However, as these measures were gradually relaxed in subsequent years (2021 and 2022), a slight increase in cases was observed (Fig. 1). Similar trends have been reported in other countries, including Türkiye, Lebanon, and England [7, 17, 29], where reductions in *E. histolytica* infections were particularly notable. Others reports from England and New Zealand showed that *Cryptosporidium* cases decreased during the COVID-19 pandemic, particularly human-to-human infections [3, 24]. This decline may be attributed to COVID-19 containment measures, which inadvertently helped control GI protozoal infections, given their fecal-oral transmission route. Additionally, public health policies such as the closure of daycare centers and schools, social distancing mandates, and heightened sanitation efforts likely contributed to the decrease in cases [23, 34].

Furthermore, individual behavioral changes related to basic hygiene measures, including continuous handwashing and use of hand sanitizers, are also expected to play an important role in relation to other parasites that cause diarrhea [4, 8]. However, it is important to consider potential biases in case estimates. Factors such as limitations in detection methods, the presence of asymptomatic infections, early diagnosis capabilities, the sensitivity of diagnostic techniques, and a general decline in testing due to the overwhelming focus on COVID-19 may have influenced reported infection rates [29].

The analysis of GI protozoal infections during the early phases of the COVID-19 pandemic revealed a general downward trend in case numbers (Fig. 2). This decline is likely associated with the widespread dissemination of precautionary measures aimed at preventing COVID-19 transmission. It has been demonstrated that exogenous health crises can promote the adoption of preventive behaviors, such as improved hygiene practices, including frequent handwashing and the use of hand sanitizers [4]. Following the official declaration of the COVID-19 pandemic by the WHO, the implementation of Phase 1 containment measures in Mexico, and the indefinite closure of schools, a more pronounced reduction in cases was observed, particularly in March and April 2020 (Fig. 2). These findings align with previous studies indicating that public health policies, such as daycare centers and school closures and social distancing, significantly reduce the incidence of GI infections caused by protozoan parasites [23, 34].

The observed decrease in the number of reported parasitic infections during the COVID-19 pandemic may not necessarily reflect a true reduction in incidence. Instead, it could be partially explained by an underestimation of cases due to reduced access to healthcare services, underreporting, and changes in health-seeking behavior. During lockdowns and periods of healthcare system strain, individuals with mild or asymptomatic infections may have avoided or been unable to access medical care, leading to fewer diagnoses and notifications. As the data show (Figs. 2A–2D), during the 2nd and 3rd waves of COVID-19, the incidence of parasitic protozoan cases decreased (Fig. 2D), while COVID-19 cases were increasing (Fig. 2A–2C). For instance, a study analyzing data from Aragón, Spain, observed a significant reduction in reported cases of *G. lamblia* and *Cryptosporidium spp*. infections in children during 2020. The authors suggest that this decrease may be related to the pandemic’s impact on healthcare services and patient behavior, leading to fewer diagnoses and notifications [25]. Similarly, research conducted in a tertiary-care hospital and a referral center for tropical diseases in Madrid reported a decline in the prevalence and incidence of enteric protozoa, including *G. lamblia* and *Cryptosporidium spp*., during the pandemic years [30].

The most notable decline in GI protozoal infections occurred during the peak implementation of COVID-19 containment measures in Mexico, particularly in May 2020, when strict lockdowns and closures of non-essential businesses were enforced (Fig. 2) [28, 36]. This downward trend reversed with the reopening of commercial activities in June, which coincided with a resurgence of COVID-19 cases and a subsequent increase in protozoal infections, underscoring the importance of sustained preventive efforts [4, 29].

Although partial restrictions were reintroduced in mid-July, only a temporary decrease in infection rates was observed, followed by a rebound and relative stabilization from September 2020 to January 2021 (Fig. 2). The gradual relaxation of health measures during the vaccine rollout in early 2021 was again accompanied by a rise in protozoal infections, with June 2021 marking the lowest observed post-pandemic reductions. These patterns reflect the strong association between public health policies and the incidence of GI parasitic diseases [9, 29].

### Analysis of changes in age and sex distribution

The analysis of age and sex distribution patterns of GI protozoal infections in Mexico, before and after the COVID-19 pandemic, revealed no significant shifts in the most affected demographic groups. In both periods, infections were more prevalent among young children, particularly those under five years of age, while adults aged 25 to 44 showed the lowest incidence (Figs. S1A–S1C). A consistently higher infection rate was observed in females across all age groups (Figs. S1D), consistent with previous studies in Mexico and other countries [18, 20, 22]. Other studies have similarly reported no significant post-pandemic changes in these patterns [17, 29].

The higher incidence among females, regardless of age, may be explained by traditional gender roles in many cultures, where women are more frequently responsible for childcare (*e.g.*, diaper use), food handling, and household sanitation, which persisted during the pandemic. This aligns with previous research on risk factors associated with protozoal infections [13, 18, 40].

### Seasonal and environmental factors affecting incidence

The monthly and yearly incidence analysis for the pre-pandemic (2017–2019) and post-pandemic (2020–2022) periods showed that the highest number of new cases of amebiasis, giardiasis, and other intestinal infections occurred during spring and summer, coinciding with Mexico’s hot and humid climate as well as vacation periods (Fig. 4). In contrast, the lowest incidence was observed during the cold and dry months of autumn and winter. These findings align with previous studies indicating that environmental conditions significantly influence parasite prevalence [18, 22, 40], with temperature increases correlating with higher protozoal infection rates.

### Sociodemographic factors and regional variability

To assess the impact of sociodemographic factors, we analyzed the average incidence by state in the pre-pandemic and post-pandemic periods, estimating the relative change, with respect to pre-pandemic years, with percentage reduction for each parasitic disease.

During the analyzed period, it was observed that the states with the greatest reductions in the incidence of GI diseases caused by protozoa also showed improvements in basic sanitation indicators, such as access to drinking water, availability of sewage systems, and a decrease in homes with earthen floors. Notable exceptions include Aguascalientes, Mexico City, and Coahuila, which already had more than 98% of their population with access to drinking water before the pandemic, and Yucatán, which did not show a significant reduction in homes with earthen floors (Fig. 6).

In contrast, the states with less reduction in the incidence of these diseases showed an increase in the population without access to drinking water (except for Guerrero, Campeche, and Baja California, which already had more than 7% of their population without access to drinking water before the pandemic), without sewage systems (except for Oaxaca and Baja California), and with earthen floors (except for Tabasco and Veracruz) (Fig. 6). These findings are consistent with previous studies that have identified a significant association between lack of access to sanitation facilities and a higher risk of GI protozoal infections [6, 38].

Similarly, socioeconomic conditions such as overcrowding and poverty have been associated with a higher incidence of GI infections caused by protozoa [39, 43]. Our data reflect this trend, as states with greater reductions in the incidence of amebiasis also showed decreases in overcrowding levels (except Coahuila, Tamaulipas, and Aguascalientes) and poverty (except Yucatán, Mexico City, and Aguascalientes) (Fig. 6). On the other hand, states with less reduction in the incidence of amebiasis showed a decrease in overcrowding (except Baja California and Sonora) (Fig. 6), but, interestingly, a greater reduction in poverty levels (except Sonora) (Fig. 6).

This apparent contradiction could be explained by the fact that, although these states have historically shown high levels of poverty due to various sociocultural factors, recent public policies have focused their efforts on mitigating this phenomenon [10, 11]. As a result, the population living in poverty has decreased overall; however, these states still host a larger proportion of people living in precarious conditions.

In addition to the previously mentioned factors, there is clear evidence that other social determinants, such as nutrition, access to healthcare services, and employment, significantly influence the incidence of intestinal infections caused by parasitic protozoa [1, 31, 37, 42]. Studies have shown that malnutrition and food insecurity increase vulnerability to these infections due to the weakening of the immune system and exposure to contaminated food and water. For example, a study on children in rural areas of Mexico found an association between energy intake and the presence of specific intestinal parasitic infections [46].

Our data indicate that the states least affected by lack of access to healthcare services (except for San Luis Potosí and Morelos), access to nutritious and quality food (except for Aguascalientes, Mexico City, and Yucatán), and lack of paid employment (except for Aguascalientes, Tamaulipas, and San Luis Potosí) present lower incidence rates of intestinal diseases caused by parasitic protozoa (Fig. 6).

In contrast, the states with the highest incidence of these diseases face deficiencies in access to healthcare services (except for Baja California and Sonora), access to nutritious and quality food (except for Guanajuato), and lack of paid employment (except for Guanajuato and Baja California) (Fig. 6). These results suggest that improvements in nutrition, access to healthcare, and employment could significantly contribute to reducing the incidence of intestinal infections caused by parasitic protozoa [1, 27, 31].

### Influence of public health measures during the COVID-19 pandemic

During the influenza A (H1N1) pandemic, it was observed that certain preventive measures helped reduce the transmission of various infectious diseases. In this context, we focused on analyzing how public health measures implemented during the COVID-19 pandemic – such as lockdowns, social distancing, mask use, and increased personal hygiene – may have been related to a reduction in the number of cases of intestinal diseases caused by parasitic protozoa.

Our data support this hypothesis, showing that states with a greater reduction in cases of intestinal diseases caused by parasitic protozoa implemented stricter public health measures more rigorously, such as school closures (Fig. 7A), international travel restrictions (Fig. 7D), stay-at-home orders (Fig. 7F), and the closure of non-essential businesses (Fig. 7B). These practices limited interpersonal contact and exposure to sources of infection, thereby reducing the spread of these pathogens.

In contrast, in states with a smaller reduction in GI protozoal diseases incidence, fewer pandemic-related public health measures were implemented, including more lenient international travel restrictions, fewer workplace closures, and lower compliance with stay-at-home orders and event cancellations (Fig. 7). These data coincide with those previously reported in other studies, where restriction measures contributed to reducing the number of cases [3, 4, 23, 35].

Taken together, socioeconomic factors such as poverty, lack of adequate sanitation and water treatment systems, illiteracy, and poor hygienic practices have been identified as significant risk factors associated with *Giardia* infection in different communities [14, 20]. This is especially important during pandemic periods, where restriction policies, social contact dynamics, together with the increase in hygiene measures, maximize the effect of these factors [4, 21, 23].

### Implications and public health considerations

Sociodemographic and economic factors are crucial predictors of parasitic disease incidence. Addressing these factors is essential for reducing the risk of GI protozoal infections, particularly in contexts where healthcare services are limited, such as during pandemics [4, 8, 20, 41]. Notably, many public health measures implemented to mitigate COVID-19 transmission – such as improved hygiene and handwashing – also contributed to reducing diarrheal diseases caused by protozoan parasites. Similar effects were observed during the 2009 H1N1 influenza pandemic in Mexico, where enhanced hygiene protocols played a decisive role in controlling parasitic infections [4, 23, 29].

Prospective epidemiological studies are essential to further elucidate these relationships and optimize health interventions. Targeted efforts should focus on eliminating risk factors and strengthening public health programs, particularly in vulnerable regions where socioeconomic disparities remain a key determinant of infection risk.

## Conclusion

In this study, we analyzed changes in the incidence of amoebiasis, giardiasis, and other GI protozoan parasites before and during the COVID-19 pandemic. We highlight the broad health implications and valuable lessons derived from the ripple effects of the COVID-19 pandemic on various aspects of healthcare. The data demonstrate a decline in the prevalence of these GI infections, with no notable changes in frequency by sex and age. However, a higher incidence was observed during months with elevated temperature and humidity.

Sociodemographic variables such as poverty, limited access to healthcare services, inadequate nutrition and food quality, unemployment, and overcrowded living conditions were associated with increased infection rates. Additionally, the data show the impact of public health interventions on disease control, demonstrating that measures implemented to contain COVID-19 (such as international travel restrictions, closure of non-essential businesses, cancellation of public events, stay-at-home orders, and promotion of handwashing and disinfection) also contributed to the reduction of intestinal protozoal infections.

The persistent burden of protozoal infections across both study periods highlights the urgent need to improve sanitation, personal hygiene, and public health education. Strengthening these factors is essential for reducing the high prevalence of protozoal infections, particularly in developing countries, where limited resources and socioeconomic disparities continue to hinder effective disease control.

## Supplementary material

The supplementary material for this article is available at https://www.parasite-journal.org/10.1051/parasite/2025049/olm.
